# Cadmium Accumulation in the Goat Liver and Kidney Is Partially Promoted by the Upregulation of Metal Transporter Genes

**DOI:** 10.3390/ani12111408

**Published:** 2022-05-30

**Authors:** Kefyalew Gebeyew, Chunyu Jiang, Qinghua Gao, Liping Zhang, Hanhua Zhu, Yushi Tian, Qi Wang, Yuqing Wei, Zhiliang Tan, Xuefeng Han

**Affiliations:** 1 Key Laboratory of Animal Husbandry Science and Technology of Xinjiang Production and Construction Corps, College of Animal Science, Tarim University, Alar 843300, China; kefyalewgebeyew@mails.ucas.edu.cn (K.G.); jcydky@126.com (C.J.); gqhdky@126.com (Q.G.); 2 CAS Key Laboratory for Agro-Ecological Processes in Subtropical Region, National Engineering Laboratory for Pollution Control and Waste Utilization in Livestock and Poultry Production, South-Central Experimental Station of Animal Nutrition and Feed Science in Ministry of Agriculture, Institute of Subtropical Agriculture, The Chinese Academy of Sciences, Changsha 410125, China; zlpeap@isa.ac.cn (L.Z.); hhzhu@isa.ac.cn (H.Z.); christgsy@hotmail.com (Y.T.); 18692266418@163.com (Q.W.); wyq523990006@163.com (Y.W.); zltan@isa.ac.cn (Z.T.)

**Keywords:** cadmium, gene expression, goat, liver, kidney, metal transporter, accumulation

## Abstract

**Simple Summary:**

The pattern of cadmium (Cd) accumulation and the role of metal transport in tissues’ cadmium deposition using Cd from an organic source are not well-clarified in ruminants. The present study results clearly showed that dietary Cd exhibited different deposition rates between goat liver, kidney, and muscle. Cd concentration in the liver, kidney, and muscle of goats fed 1.07 mg Cd/kg of dry matter (DM) for 60 days remained within the scope of (1.0 mg/kg) the food safety standards of China (GB 2762-2017) and the European Commission Regulation 1881/2006 (amended by No. 629/2008). Dietary Cd promoted the expression of metal transporter genes in the liver and kidneys of goats. *DMT1*, *ZIP8*, and *ZIP14* might play an imperative role in the uptake of Cd in the goat liver and kidney but not in the muscle.

**Abstract:**

Metal transporters, including divalent metal-ion transporter-1 (DMT1), Zrt-/Irt-like protein 8 and 14 (ZIP8 and ZIP14), and ferroportin-1 (FPN1), reportedly participate in cellular cadmium (Cd) uptake, but those in farm animals remain unclarified. This study aimed to examine the growth, plasma biochemical indices, Cd accumulation, and expression of metal transporter genes in the liver, kidney, and muscle of goats exposed to rice paddies contaminated with different levels of Cd. Twenty-four goats were randomly assigned across three dietary treatments: 0.23, 0.63, and 1.07 mg of Cd/kg of dry matter (DM) for 60 days. The results showed that dietary Cd exposure increased (*p* < 0.05) both Cd accumulation and the mRNA expressions of metal transporter genes (*DMT1*, *ZIP*, and *FPN1*) in the liver and kidney but not in the muscle, suggesting dietary Cd exhibited different deposition rates between goat liver, kidney, and muscle. These outcomes suggest that high levels of dietary Cd stimulated the expression of metal transporter genes and thereby enhanced the uptake and accumulation of Cd in the goat liver and kidney. As such, higher Cd concentrations in the liver and kidney observed with Cd diets could be partly explained by upregulation of metal transport genes expression.

## 1. Introduction

Cadmium (Cd) is a worldwide contaminant heavy metal that has been classified as an environmental hazard and is present in various agricultural crops [1] and animal tissues [2]. Livestock exposed to plants with either high or low Cd contamination accumulate Cd within their tissues, which results in damage to the physiological and biochemical functions of various organs [3,4]. The adverse effects of Cd in the organs are the changes in the production of reactive oxygen species (ROS) and modulation of metals’ homeostasis [3,5]. Previous studies have shown that Cd accumulates in almost all animal tissues, particularly in hyperaccumulators such as the liver and kidney [6]. Hence, residual Cd in meat and offal is one of the main sources of Cd in the food chain and may be a risk to the health of humans consuming high levels of meat and offal derived from livestock exposed to Cd in contaminated areas [7,8]. To this end, countries have set a regulation for a Cd limit in the diet and animal products to protect human health. In this regard, the Chinese National Food Safety Standard (GB 2762-2017) and European commission regulation 1881/2006 (amended by No. 629/2008) have set the maximum concentration of Cd in the kidneys, liver, and muscle of cattle for human consumption at 1.0, 0.5, and 0.05 mg/kg wet weight, respectively.

Dietary Cd can be taken up by the animal body via the same pathways as essential metals are. The absorption and transport of Cd in various tissues require the participation of different metal transporters, and multiple transporters involved in the uptake of Cd by mammalian cells have been identified [9,10]. It has been reported that divalent metal transporter 1 (DMT1), which was initially known as a ferrous iron transporter in the intestine, can transport several divalent metal ions, such as Cd and Fe [11]. Some studies have shown the vital function of DMT1 in Cd absorption in the gastrointestinal tract [11,12]. Mammalian ZIP8 (SLC39A8) and ZIP14 (SLC39A14) are transmembrane proteins that belong to the Zrt-/Irt-like protein (ZIP) family, which consists of 14 members that share considerable homology [13]. Previous studies have identified ZIP8 and ZIP14 as zinc transporters, and these proteins have also been shown to participate in the uptake of Cd [14]. Enhancing the expression of ZIP8 and ZIP14 in Xenopus oocytes results in improved Cd uptake, which suggests that ZIP8 and ZIP14 are responsible for Cd uptake [15].

Ferroportin 1 (FPN1) is recognized as a mammalian iron exporter and is involved in the transport of iron and other metals [16]. The mRNA expression level of FPN1 dose- and time-dependently increases when J774 macrophage cells are treated with different concentrations of CdCl2 (5–25 μM) for 8 h [17]. The oral administration of Cd enhances the bodily burden of Cd and the mRNA expression of DMT1 and FPN1 in the duodenum of rats fed a Fe-deficient diet and repressed under Fe-sufficient conditions [12]. Findings from various in vivo and in vitro studies have highlighted the role of metal transporters, particularly DMT1, ZIP8, and ZIP14, in Cd uptake in different tissues and cell types under particular physiological conditions [10,11,12,13,14,15,16,17,18]. The involvement of these metal transporters in Cd accumulation in the specific tissue of farm animals has not yet been reported.

The uptake, absorption, and accumulation of Cd in different target tissues have been extensively investigated in animals not used for human consumption and treated with a relatively high dosage of Cd from an inorganic source [19,20,21]. However, these results may not reflect the real condition of animals exposed to Cd because their absorption characteristics are relatively lower than those of organic compounds incorporating Cd. In addition to that, ruminants accumulate Cd at a different rate than monogastric animals because ruminant animals possess phytase-producing flora for digesting phytic acid, which sequesters Cd and increases its availability for absorption [22]. Thus, we hypothesized that metal transporters might be partially or fully involved in the uptake of Cd in various tissues of goats. Our objective was to investigate the growth, blood biochemical indices, and gene expression of *DMT1*, *ZIP8*, *ZIP14*, and *FPN1* in the liver, kidney, and muscle of goats exposed to rice paddies contaminated with different levels of Cd.

## 2. Materials and Methods

### 2.1. Animals and Experimental Design

All experimental animal procedures were performed following the protocols approved by the Animal Care and Use Committee of the Institute of Subtropical Agriculture, Chinese Academy of Sciences, Changsha, China (Permit No. ISA000235). Twenty-four Xiangdong male black goats with initial body weights (IBWs) of 15.27 ± 1.85 kg and aged approximately nine months were used in this study. All the goats were received from a local goat farm after having their health and wellbeing checked. The goats were randomly allocated to one of three dietary treatments: control group (CON; a basal diet containing 0.23 mg of Cd/kg of DM), moderate Cd group (MCd; a basal diet supplemented with 0.63 mg of Cd/kg of DM), and high Cd group (HCd; a basal diet supplemented with 1.07 mg of Cd/kg of DM). The moderate Cd group was designed following the results of a previous study in our companion team using 0.60 mg of Cd/kg of Cd-contaminated rice paddy as a moderate dose [23]. The animals were housed individually in stainless steel cages and fed a diet containing a concentrate and forage twice daily. The roughage was chopped rice straw, and the concentrate was mainly composed of rice paddies. The ratio of concentrate to roughage was 55:45. The same rice paddy variety with different Cd contents was used to prepare three diets with different Cd levels. The Cd-contaminated rice paddy was provided by the Beishan test station (Changsha, China), which is located in a contaminated zone. The quantity of Cd in the rice paddy was determined before its use in this study. The diets were prepared according to the nutritional requirements for growing goats [24], and the detailed ingredients and nutritional composition of the diets are shown in Table 1. The feeding trial period consisted of 60 days for the actual trial and 10 days of adaptation. The supplied feed was adjusted for each group in the morning according to dry matter intake (DMI) on the previous day to ensure at least 5% refusals.

### 2.2. Growth Performance and Plasma Biochemical Indices

The final body weight (FBW) of each goat was determined on day sixty. The average daily feed intake (ADFI) and the average daily gain (ADG) were calculated as described by Wang et al. [25]. At the end of the trial, blood samples were collected from the jugular vein before the morning feeding using 5 mL vacuum blood collection tubes with sodium heparin as an anticoagulant (Aosaite Medical Devices Co., LTD, Heze, China). Subsequently, the blood was centrifuged (3000× *g*) at 4 °C for 10 min to obtain plasma. All plasma samples were free of hemolysis and frozen at −20 °C until analysis. Plasma biochemical indices, including aspartate aminotransferase (AST), alanine aminotransferase (ALT), gamma-glutamyltransferase (GGT), alkaline phosphatase (ALP), lactate dehydrogenase (LDH), total protein (TP), creatinine (CRET), blood urea nitrogen (BUN), total cholesterol (TC), triglyceride (TG), high-density lipoprotein cholesterol (HDL-C), low-density lipoprotein cholesterol (LDL-C), and glucose (GLC) were determined using an automatic biochemical analyzer (Cobas c311, Roche, Basel, Switzerland) and commercial analytical kits from Roche (Shanghai, China) as described elsewhere [26].

### 2.3. Determination of the Cd and Fe Concentration in Tissues

All goats were killed by bleeding the jugular vein by a registered veterinarian after 16 h of fasting, and the kidneys, liver, and longissimus dorsi muscle of each animal were rapidly separated. The samples from these tissues were separated into two parts. One part was stored at −80 °C after flash freezing in liquid nitrogen for RNA extraction, and the other part was stored at −20 °C for Cd analysis. The kidney, liver, and muscle samples were freeze-dried and then ground into a powder to determine the Cd concentrations. The kidney cortex and medulla were uniformly mixed to avoid differences in the Cd and Fe concentrations, and the concentrations are expressed for the whole organ. The weighed samples were digested with concentrated nitric acid (HNO_3_) and diluted with double distilled water, as described by Yao et al. [27] with minor modifications. Briefly, the weighed samples were transferred to triangular flasks containing 15 mL of acid mixture (HNO_3_:HClO_4_ = 5:1(*v/v*)). The flasks were covered with funnels and left at room temperature for cold digestion overnight. Then, the digestion flasks were placed on the electric heating panel. Digestions were initially performed at 120 °C until the solutions turned yellow transparent and then, the temperature was increased to 180 °C for 30 min. The temperature was further increased to 260 °C until the solutions turned colorless. Then, the funnels were removed from the flasks, and the solutions were almost completely evaporated. After cooling to room temperature, the residues were dissolved in 1% (v/v) HNO_3_ and transferred into a 10 mL colorimetric tube, and the final volume was made up to 10 mL. After filtration, the concentrations of Cd and Fe in the digested samples were determined by inductively coupled plasma mass spectrometry (Agilent Technologies, 7700 series ICP-MS, USA). The blank digests without samples and the reference material were also treated using the same protocol to confirm the accuracy of the analytical results. The concentrations of Cd and Fe were calculated on wet weight basis, and the results are expressed as milligrams per kilogram.

### 2.4. RNA Extraction and cDNA Synthesis

Total RNA from the kidney, liver, and muscle samples was isolated using the Trizol reagent (Invitrogen, CA, USA) according to the manufacturer’s protocol. The quantity and purity of total RNA were measured using a NanoDrop 2000 spectrophotometer (Thermo Fisher Scientific, Waltham, MA, USA). The total RNA integrity was assessed by running an aliquot of the RNA sample on a denaturing agarose gel. The cDNA was synthesized using a PrimeScript RT Reagent Kit with a gDNA Eraser and a Random RT Primer Mix (Takara, Dalian, China) based on the manufacturer’s instructions, and stored at −20 °C until analysis [28].

### 2.5. Gene Expression Profile

The metal transporter genes investigated in this study were DMT1, ZIP8, ZIP14, and FPN1. As reference genes, expression of *β-actin (ACTB)* and glyceraldehyde-3-phosphate dehydrogenase (*GAPDH*) were analyzed. The stability of the reference genes was assessed using BestKeeper, Genorm, and NormFinder tools [29]. The two reference genes were found as stable reference gene across experimental treatment and were used to normalize the data. The primer sequences of the targeted genes and internal references are listed in Table 2, and all the primers were synthesized by Sangon Biotech Co., Ltd. (Shanghai, China) (Supplementary S1). The amplification efficiency of each gene in this study was computed by constructing a standard curve through serial dilutions of cDNA. All primers showed efficiency between 90 and 110%, and correlation coefficients were close to 1.0. Real-time PCR analysis of the expression of the above-mentioned genes was performed on a Lightcycler 480 II System (Roche, Basel, Switzerland) with a SYBR Premix Ex Taq II 98 (Tli RNaseH Plus) detection kit (Takara, Kusatsu, Japan). The thermal cycles were as follows: initial denaturation at 95 °C for 5 min; 40 cycles of 95 °C for 10 s, 60 °C for 30 s, and 72 °C for 30 s; and a final extension at 72 °C for 5 min. The average cycle threshold values were calculated using the 2^−ΔΔCt^ method [30].

### 2.6. Statistical Analysis

The SPSS version 23 (SPSS Inc., Chicago, IL, USA) and Origin 2020b statistical analysis tools were used to analyze the data. The data were subjected to one-way analysis of variance (ANOVA) after checking for normality and homoscedasticity of variance using Shapiro–Wilk and Levene’s tests. Tukey’s post hoc tests were used to determine the differences between treatment means, a significant difference was regarded as *p* < 0.05, and trends were recognized at 0.05 ≤ *p* < 0.1. The results are expressed as the means ± standard errors of the mean (SEMs).

## 3. Results

### 3.1. Effects of Dietary Cd on Growth Performance and Plasma Biochemical Indices

**Figure 1:**
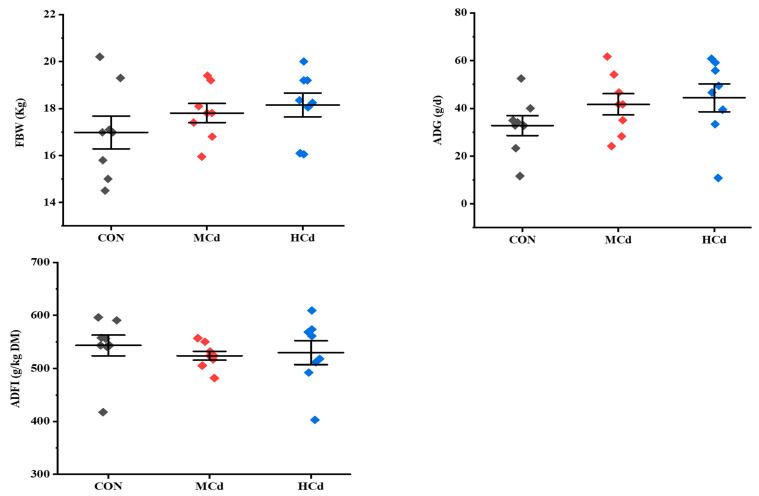
Effects of dietary cadmium levels on FBW, ADG, and ADFI of goats. FBW: final bodyweight gain; ADG: average daily gain; ADFI: average daily feed intake; treatments: CON, MCd, HCd: 0.23, 0.63, and 1.07 mg of Cd/kg of diet DM, respectively.

**Figure 2:**
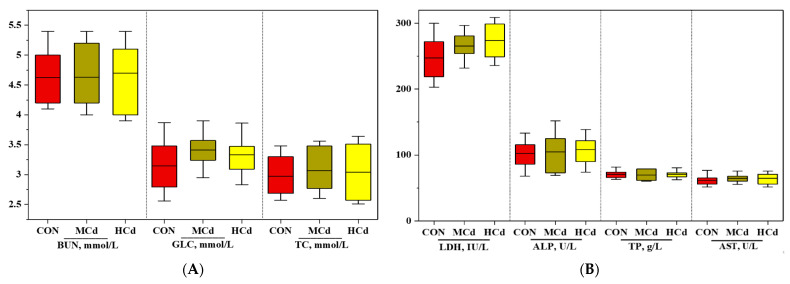

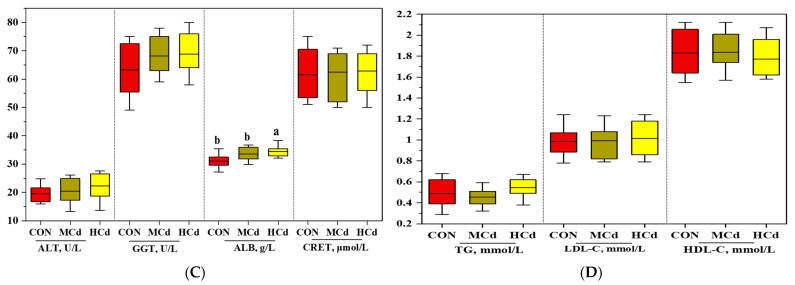
Effects of dietary cadmium levels on blood biochemical indices (**A**–**D**) of goats. The values are expressed as the mean ± SEM. One-way ANOVA was used, followed by post hoc Tukey’s test. Different letters (a, b) indicate significant difference a significant difference among the three dietary treatments at *p* < 0.05. ALT: alanine aminotransferase; AST: aspartate aminotransferase; GGT: gamma-glutamyl transferase; ALP: alkaline phosphatase; LDH: lactate dehydrogenase; ALB: albumin; CRET: creatinine; TC: total cholesterol; TG: triglyceride; LDLC: low-density lipoprotein cholesterol; HDLC: high-density lipoprotein cholesterol; BUN: blood urea nitrogen; GLC: glucose. Treatment: CON, MCd, HCd: 0.23, 0.63, and 1.07 mg of Cd/kg of diet DM, respectively.

The growth performance and plasma biochemical indices are shown in Figure 1 and Figure 2. No significant differences (*p* > 0.05) in FBW, ADG, and ADFI were observed among the treatment groups. Similarly, increasing the Cd level in the diet did not affect (*p* > 0.05) the plasma concentrations of ALT, AST, GGT, ALP, LDH, TP, CRET BUN, TG, TC, LDL-C, HDL-C, and GLC. However, the plasma ALB concentration increased with increases in dietary Cd levels (*p* < 0.05).

### 3.2. Effects of Dietary Cd on the Cd and Fe Concentrations in the Liver, Kidney and Muscle Tissues

**Figure 3:**
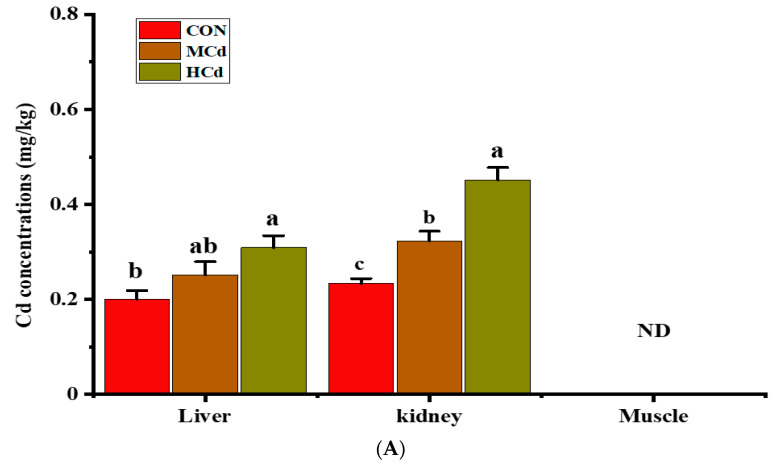

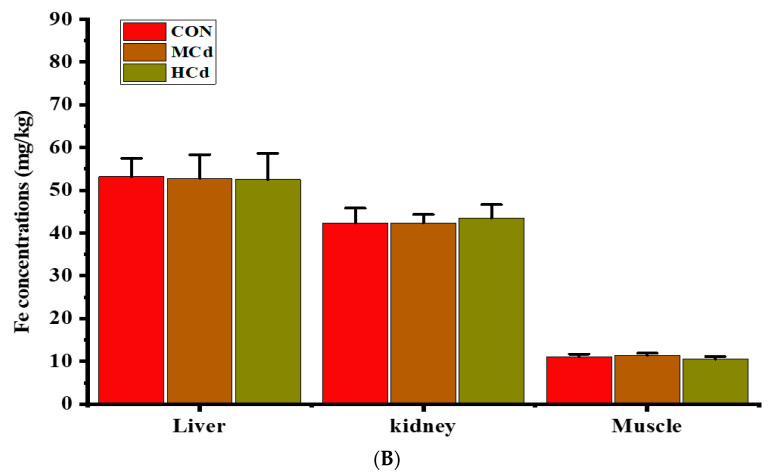
Effects of dietary cadmium levels on (**A**) cadmium and (**B**) iron concentrations in the liver, kidney, and muscle of goats. Different letters (a, b, c) indicate significant difference a significant difference among the three dietary treatments at *p* < 0.05. Values are expressed as the mean ± SEM. One-way ANOVA was used, followed by post hoc Tukey’s test. *n* = 8. ND = Not detected (below the instrument detection limits). Treatment: CON, MCd, HCd: 0.23, 0.63, and 1.07 mg of Cd/kg of diet DM, respectively.

The concentrations of Cd and Fe in the liver, kidney, and muscle are shown in Figure 3. In the kidney, Cd concentration was greater (*p* < 0.05) in the HCd group than that in the CON group but remained within the scope (1.0 mg/kg) of the food safety standards of China (GB 2762-2017) and the European Commission Regulation 1881/2006 (amended by No. 629/2008). The liver Cd concentration in the HCd group was remarkably higher (*p* < 0.05) than that in the control group (0.30 mg/kg vs. 0.20 mg/kg), and both values were within the scope (0.5 mg/kg) of the food safety standard of China and the EU. In all groups, the Cd concentration in the muscle was below the instrument detection limit. No differences (*p* > 0.05) in the concentrations of Fe in the liver, kidney, and muscle were found among the treatments.

### 3.3. Effects of Dietary Cd on the Expression of Genes Involved in Metal Transport

**Figure 4:**
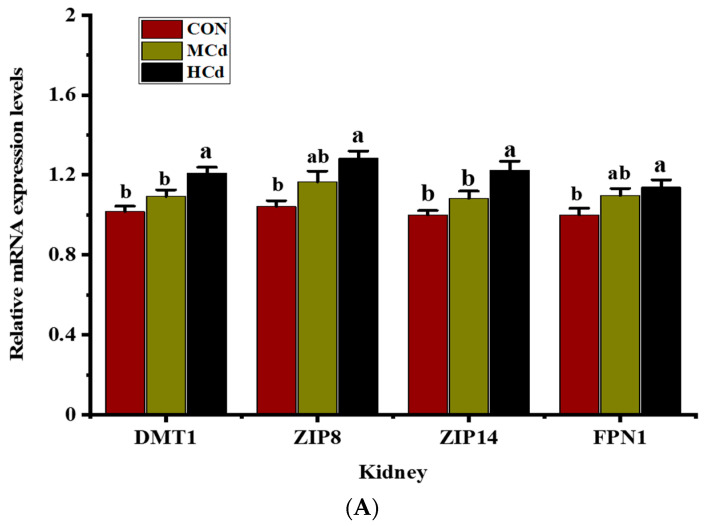

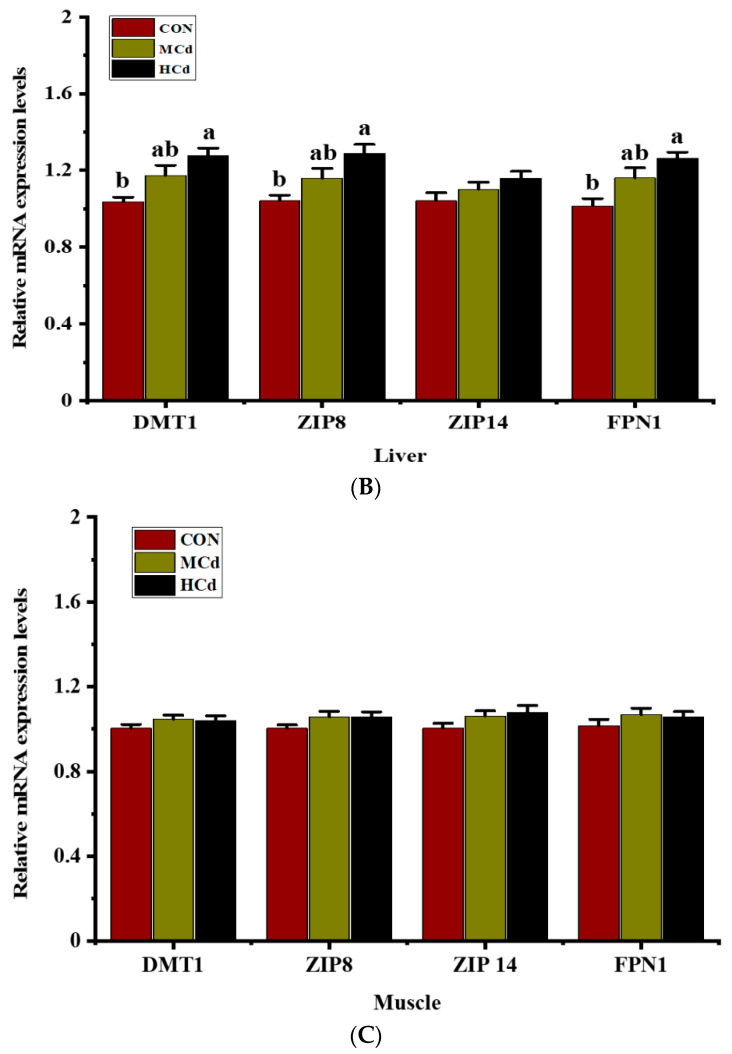
Effects of dietary Cd levels on the gene expressions of *DMT1, ZIP8, ZIP14* and *FPN1* in the (**A**) kidney, (**B**) liver, and (**C**) muscle of goats. Different letters (a, b) indicate significant difference a significant difference among the three dietary treatments at *p* < 0.05. Values are expressed as the mean ± SEM. One-way ANOVA was used, followed by post hoc Tukey’s test. ND = Not detected. Treatment: CON, MCd, HCd: 0.23, 0.63, and 1.07 mg of Cd/kg of diet DM, respectively. DMT1: divalent metal-ion transporter-1; ZIP8: Zrt-/Irt-like protein 8; ZIP14: Zrt-/Irt-like protein 14; FPN1: ferroportin-1.

The effects of dietary Cd levels on the mRNA expression of four genes involved in metal transport in the liver, kidney, and muscle are shown in Figure 4. Compared with those in the CON group, goats fed a diet with high Cd levels presented higher (*p* < 0.05) mRNA expression levels of DMT1, ZIP8, ZIP14, and FPN1 in the kidney. Similarly, the mRNA expression levels of DMT1, ZIP8, and FPN1 in the liver significantly increased (*p* < 0.05) as the dietary Cd levels increased from 0.23 to 1.07 mg/kg of DM. In addition, the ZIP14 mRNA level in the liver of the MCd and HCd groups was higher than that in the control group, although the difference did not reach a significant level (*p* > 0.05). No differences (*p* > 0.05) in the mRNA expression levels of DMT1, ZIP8, ZIP14, and FPN1 in muscle was found among the treatments.

## 4. Discussion

Cadmium has become an occupational and environmental pollutant due to its extensive and continued use in industry and agriculture [31]. Cd can exert numerous deleterious effects on various organ functions when it enters the animal body. Once ingested by an animal, Cd is efficiently retained in different organs. It has been reported that Cd could accumulate on various tissues of farmed ruminants [2]. The accumulation of Cd in different tissues requires the involvement of metal transporters. Several transporters that play a significant role in Cd accumulation, such as DMT1, ZIP, and FPN, have been identified in mammalian cells [17,18], but the involvement of metal transport in Cd uptake in livestock has not been reported. In addition, the adverse effects of Cd on the tissue and transport mechanism have been examined using a relatively high dosage of Cd from an inorganic source. These results may not reflect the actual condition of animals exposed to Cd. Thus, we used Cd-contaminated rice paddies to investigate the effects of dietary Cd levels on the growth performance, plasma biochemical indices of goats, and the expression of key metal transporter genes in the liver, kidney, and muscle of these animals.

In the present study, neither growth performance nor ADFI were affected by increased dietary Cd levels. These results agree with those of previous reports [32,33]. However, several studies have reported that the growth performance of pigs declined with increases in dietary Cd concentration to 2.5 mg/kg [2]. The discrepancy in the results might be due to the Cd level in the diet. In this sense, Lane et al. [34] reported that growth performance and milk production are affected by long-term daily intake of dietary Cd higher than 30 mg/kg. These results indicate that the Cd concentration in feed is the primary factor producing growth performance defects.

Plasma metabolite changes may be symptomatic of an animal’s physiologic state because they are intermediary metabolism products [35]. Plasma AST, ALT, LDH, GGT, TP, CRET, and BUN have been used to assess the liver injury induced by Cd exposure [36,37]. Exposure of rats to a diet containing Cd-contaminated radish bulb (1.1 mg Cd/g of diet) increased plasma AST, ALT, LDH, and CRET by the 12th week but not by the fourth week [32], indicating liver injury or the loss of cellular integrity and leakage of the hepatic membrane. In our results, plasma AST, ALT, LDH, GGT, TP, CRET, and BUN were unaffected by increases in dietary Cd levels, suggesting that liver function was intact under the experimental conditions. Exposure to a Cd dose of up to 1.07 mg/kg could induce adaptive responses and compensatory mechanisms to protect the goat liver from Cd-induced injury. A similar adaptive response was observed in rats chronically exposed to a low dose of CdCl2 (4.8 mg CdCl2/L) for a year [38]. Thus, the Cd level and duration of exposure were the primary determinants of the induction of tissue damage by Cd.

In the present study, increases in dietary Cd levels to 1.07 mg Cd/kg of DM did not affect plasma TC, TG, LDL-C, HDL-C, and GLC concentrations, suggesting that blood lipid metabolism was unaffected by dietary Cd levels. Chronic exposure (for 370 days) of rats to a low dose of CdCl2 (4.8 mg CdCl2/L) dissolved in water does not induce significant changes in plasma TC, TG, and GLC [38]. However, some studies involving the administration of Cd in animal models have demonstrated changes in TC, TG, LDL-C, and HDL-C profiles. Olisekodiaka et al. [39] showed that intraperitoneal administration of 1 mg of Cd/kg of BW to male albino rats for four weeks resulted in significant increases in plasma TC, TG, and LDL-C, and a decrease in HDL-C. Another experiment using male rats demonstrated that exposure to 5 and 50 mg/L of Cd in drinking water for six months resulted in dose-dependent reductions in serum LDL-C and HDL-C with no effects on TC levels [40]. The discrepancy in lipid profile changes may result from differences in Cd dose, source, treatment regimen, and duration of exposure. Considering the available data in the literature, the small gaps in dietary Cd levels between the three groups could be a reason for the lack of significant effects on blood lipid metabolism observed in the present study.

The concentration of plasma ALB in response to Cd exposure was inconsistent among the studies, which may be associated with the dose of Cd, protein metabolism, and source of Cd. A decrease in the plasma ALB levels was observed in rats chronically exposed to a low dose of CdCl2 (4.8 mg CdCl2/L) [38]. Conversely, an increase in serum ALB has been observed in rabbits following the subcutaneous injection of CdCl2 at a dose of 3 mg/kg of BW [41]. A high plasma ALB concentration has been reported in occupationally Cd-exposed workers compared with that in non-exposed workers [42]. In this study, the plasma ALB concentration showed an increasing trend as the dietary Cd levels increased to 1.07 mg of Cd/kg of DM. These differences were probably due to species differences in protein metabolism or the adverse effects of Cd on protein metabolism. In the present study, Cd concentrations in the kidney and liver increased as the Cd levels increased to 1.07 mg Cd/kg of DM, and Cd levels in the kidney were higher than those in the liver. These results indicate that Cd accumulated in the bodies of goats.

Prankel et al. [43] have shown using a meta-analysis approach that the concentration of Cd in the diet and exposure period are the main factors that determine Cd accumulation in the kidneys. Herein, we found that Cd concentration in liver and kidney tissues was within the Chinese National Food Safety Standard (GB 2762-2017) and European Commission Regulation 1881/2006 (amended by No. 629/2008). These results suggest that Cd is less likely to join the human food chain through the liver and kidney of goats fed diets containing up to 1.07 mg/kg of Cd for two months because edible offal is widely consumed in some areas. In the present study, we found that Cd was not detected in the muscle even though Cd is accumulated in the kidney and liver. This result is also consistent with the results reported by Wilkinson et al. [2], who found that high Cd accumulation in the muscle is unlikely to occur with a daily intake of dietary Cd lower than 30 mg/kg. Conceivably, metallothioneins (MTs) could reduce the amount of free Cd accessible for muscle distribution by forming Cd-MT complexes in the liver and reabsorbed by proximal tubule epithelial cells [44]. Cd accumulation in cattle muscle has been reported elsewhere in China [7] and Belgium [45] even though the experimental approaches used in the studies were different. However, Cd accumulation in cattle muscle is not very clear because some key factors that determine the accumulation of Cd in tissues were not clearly stated in the above-mentioned studies.

Previous studies have demonstrated that the metal transport pathways used by essential metals may be involved in Cd absorption [44,46]. DMT1 is a proton-coupled metal-ion transporter that is prevalently expressed in animals and has substantial substrate specificity, preferring divalent metals, such as Fe and Cd [47]. The strong association found between Cd uptake and the expression of DMT1 in pregnant rats indicates that DMT1 plays a vital role in the uptake of Cd under physiological conditions [48]. In the present study, increases in the dietary levels of Cd resulted in increased expression of DMT1 mRNA in the liver and kidney, which suggested the participation of DMT1 in the absorption of Cd from the small intestine into the enterocyte and the subsequently transfer of Cd to the liver and kidney. These findings are supported by a previous study that DMT1 expression in the liver increases following oral Cd administration regardless of the concentration of Fe in the body [10]. In addition, no significant difference in Fe concentration was found among the treatment groups in this study. Thus, the upregulation of DMT1 mRNA is associated with marked increases in tissue Cd accumulation, which suggests that DMT1 is a strong candidate for Cd uptake in goat tissues.

ZIP14 and ZIP8, members of the ZIP family, are transmembrane proteins that can mediate Cd uptake in cells [49]. The knockdown of ZIP8 and ZIP14 in mouse proximal tubular cells causes a reduction in Cd uptake and their expression on the apical side [18]. These results suggest that the downregulation of ZIP8 and ZIP14 expression is linked to a decrease in cell Cd accumulation. Similarly, the knockdown of ZIP8 (but not ZIP14) in rat basophilic leukemic cells induces significant reductions in Cd uptake rates and ZIP8 expression [50]. In this study, increases in the concentration of Cd in the liver and kidney and the mRNA expression levels of ZIP8 and ZIP14 in the kidney were observed with increases in dietary Cd levels, which suggests that ZIP8 might play an important role in Cd accumulation in the goat liver. Additionally, the other metal transporter can compensate for the function of ZIP14 when the expression of ZIP14 is unchanged. However, Fujishiro et al. [51] indicated that the expression levels of ZIP8 and ZIP14 mRNA in Cd-resistant cells (A7 and B5) derived from embryonic fibroblasts of MT1- and MT2-knockout mouse cells are markedly suppressed, which results in a decrease in Cd accumulation. These results may show that ZIP8 and ZIP14 play a role in Cd transport in mouse cells. The discrepancy in ZIP14 expression may be due to differences in the tissue-specific roles or types of cells. Thus, the role of ZIP14 in Cd uptake should be examined in various tissues or cell types.

FPN1 is a transmembrane transporter protein expressed in tissues and cells, such as kidney proximal tubule cells and macrophages of the liver, associated with the efflux of various metal ions [52,53,54]. In this sense, Mitchell et al. [55] investigated using Xenopus laevis oocytes expressing human FPN1 and found that FPN1 does not transport Cd for vectorial reabsorption into the circulation. On the other hand, the oral administration of Cd increases the intestinal tissue accumulation of Cd and the expression of FPN1 mRNA in Fe-deficient rats compared with that in Fe supplemented-rats [12]. The authors suggested a strong association between FPN expression and the accumulation of Cd and Fe in the body. However, Cd uptake and FPN1 expression in duodenal enterocytes are unaffected by Fe deficiency in unweaned piglets and rats at postnatal days 16 and 10, respectively [56,57].

In the present study, FPN1 expression and Cd concentration were upregulated in the liver and kidney of the goats in the MCd and HCd groups, even though the liver and kidney Fe concentrations remained unchanged. Various regulatory mechanisms control the expression of FPN1 mRNA, and the observed changes in FPN1 mRNA expression could be linked to increases in the levels of the FPN1 protein or intracellular reactive oxygen species (ROS) levels induced by Cd accumulation [17]. Possibly, Cd treatment may indirectly change the intracellular Co, Zn, or Cu concentrations and thereby enhance FPN1 expression. Divalent metals could interact with Cd metabolism [58,59], and some divalent metals, such as Cu, have been shown to affect the FPN1 gene expression level [60]. To date, the function of FPN in the liver and kidney has not been well-characterized in animal model studies. Whether FPN1 is involved in Cd transport remains unclear, and more well-designed studies are needed to gain insight into the possible role of FPN1 in the transport of Cd in the liver and kidney.

## 5. Conclusions

The present results showed that Cd accumulation in the liver and kidney of goats increased as the dietary Cd levels increased from 0.23 to 1.07 mg Cd/kg of DM. Cadmium residues in the liver and kidney of goats fed a moderate- or high-Cd diet did not exceed the standard accepted level, and there was little deposition in the muscle. Dietary Cd exhibited different deposition rates between goat liver, kidney, and muscle. Dietary Cd promoted the expression of metal transporter genes, which plays an imperative role in the uptake and accumulation of Cd in the goat liver and kidney.

## Figures and Tables

**Table 1:** Ingredients and nutrient compositions of experimental diets.

Ingredient	% of DM
Paddy	33.2
Soybean meal	9.6
Wheat bran	6.0
Fat powder	3.2
Calcium carbonate	0.5
Calcium bicarbonate	1.1
Sodium chloride	0.6
Premix ^1^	1.0
Rice straw	45
Chemical Composition ^2^	% of DM
CP, %	8.8
NDF, %	47
ADF, %	33.5
Ca, %	0.47
Total P, %	0.32

^1^ Mineral and vitamins in premix per kg; Mg: 8.5 g; Zn: 4 g; Fe: 510 mg; Cu: 750 mg; Mn: 5.1 g; Se: 5 mg; I: 30 mg; Co: 11 mg; vitamin A: 104,400 IU; vitamin D: 17,500 IU vitamin E: 2200 IU. ^2^ DM: dry matter; CP: crude protein; NDF: neutral detergent fiber; ADF: acid detergent fiber; Ca: calcium; Total P: total phosphorus.

**Table 2:** Primer sequences and amplicon information.

Gene		Primers (5′~3′)	Bp	Accession	Efficiency
*DMT1* ^1^	Forward	TGCCTACAGTAATTCCTCAATTCCTCAG	169	BC113342.1	95.2
	Reverse	ATCCACAACGCTCATAAGAAGTCCTG			
*ZIP8* ^1^	Forward	TTCCAGAGATGAACGATATGCTGAGAG	115	NM_001205630.1	102.6
	Reverse	ATGAGAAGAATGGCTGTGAATCCAGTT			
*ZIP14* ^1^	Forward	AGAATGAGGAGAACGAGCAGACAGA	182	BC140602.1	108.7
	Reverse	TCCAATCGCCAGAGCTATGAAGTAGA			
*FPN1*	Forward	AGACAGAGGCAGATTAGCAGATATGAATG	134	XM_018065526.1	104.9
	Reverse	CCGAAATGAAACCACAGCCAATGAC			
*ACTB*	Forward	CTTCCAGCCTTCCTTCCTG	111	NM_001314342.1	97.1
	Reverse	ACCGTGTTGGCGTAAAGGT			
*GAPDH*	Forward	GGGTCATCATCTCTGCACCT	176	XM_005680968.3	105.2
	Reverse	GGTCATAAGTCCCTCCACGA			

^1^ Sequences are from cattle as there are no adequate goat sequences. Bp, amplicon size in base pair. DMT1: divalent metal-ion transporter-1; ZIP8: Zrt-/Irt-like protein 8 ZIP14: Zrt-/Irt-like protein 14; FPN1: ferroportin-1; ACTB: β-actin; GAPDH: glyceraldehyde-3-phosphate dehydrogenase.

## Data Availability

All data generated or analyzed during this study are available from the corresponding author upon reasonable request.

## Supplementary Materials

The supplementary materials are available online at https://www.mdpi.com/article/10.3390/ani12111408/s1.
