# Supplementary material for: Cadmium Accumulation in the Goat Liver and Kidney Is Partially Promoted by the Upregulation of Metal Transporter Genes
Source: Animals (Basel). 2022 May 30;12(11):1408. doi: 10.3390/ani12111408 (PMC9179383; doi:10.3390/ani12111408)
Supplement: Supplementary file 1 [file animals-12-01408-s001.zip › animals-1695161-SI.pdf]

Rice Paddy Collection for the Present Study

The rice paddies of the three diets in the present study were used from the same variety. The Cd-contaminated rice paddies were received from the Beishan test station, which is located in a contaminated zone. The rice paddy in the control diet came from the same town (Beishan town, Changsha county, Changsha city, Hunan province, China). The Cd levels of rice paddies were analyzed before selecting the rice paddies used for this experiment. The nutrient compositions of the three rice paddies were also analyzed, and the results showed that the nutrient compositions of the three rice paddies were similar. Therefore, only the nutrient compositions of the control diet are shown in Table 1.

Approached employed to design primers for the present study

We referred to the mRNA sequences of cattle DMT1, ZIP8, and ZIP14 to design primers in this study. The relevant work descriptions are as follows. **Firstly**, we searched the goat DMt1, ZIP8, and ZIP14 in Genbank, and there are no mRNA sequences of goat DMT1, ZIP8, and ZIP14. **Secondly**, we searched the DMT1, ZIP8, and ZIP14 mRNA sequences of cattle and sheep. Because there the no complete mRNA sequences for sheep DMT1 and ZIP14 or no mRNA sequence for sheep ZIP8, we used cattle DMt1, ZIP8, and ZIP14 sequences as templates for primer design. **Thirdly**, the conserved region is used to design primer in qPCR. The coding sequence (CDS) is the actual region of DNA that is translated to form proteins, and the CDS region is generally conserved. We used the CDS region of cattle DMT1, ZIP8, and ZIP14 for the qPCR primer design. **Fourth**, we analyzed the conservation of primers by homologous sequence alignment from cattle, sheep, humans, etc. **Fifth**, PCR amplification were performed and the results showed that the designed primers of DMT1, ZIP8, and ZIP14 were amplified using a goat template. Then after, we also checked the efficiency of all primers using a serial cDNA dilution curve. All primers showed efficiency between 90 and 110 %, and correlation coefficients were close to 1.0.
